# *Panax ginseng* Improves Functional Recovery after Contusive Spinal Cord Injury by Regulating the Inflammatory Response in Rats: An *In Vivo* Study

**DOI:** 10.1155/2015/817096

**Published:** 2015-09-15

**Authors:** Young Ock Kim, Youngkyung Kim, Koeun Lee, Sae Won Na, Seon Pyo Hong, Mariadhas Valan Arasu, Young Wook Yoon, Junesun Kim

**Affiliations:** ^1^Department of Medicinal Crop Research, Rural Development Administration, Eumseong, Chungbuk 369-873, Republic of Korea; ^2^Department of Physiology, College of Medicine, Korea University, Seoul 136-705, Republic of Korea; ^3^Rehabilitation Science Program, Department of Health Science, Korea University Graduate School, Seoul 136-701, Republic of Korea; ^4^National Rehabilitation Center, Seoul, Republic of Korea; ^5^College of Veterinary Medicine, Konkuk University, Seoul, Republic of Korea; ^6^Department of Oriental Pharmaceutical Sciences, College of Pharmacy, Kyung Hee University, Seoul 130-091, Republic of Korea; ^7^Department of Botany and Microbiology, Addiriyah Chair for Environmental Studies, College of Science, King Saud University, P.O. Box 2455, Riyadh 11451, Saudi Arabia; ^8^Department of Physical Therapy, College of Health Science, Korea University, Seoul 136-701, Republic of Korea

## Abstract

Spinal cord injury (SCI) results in permanent loss of motor function below the injured site. Neuroinflammatory reaction following SCI can aggravate neural injury and functional impairment. Ginseng is well known to possess anti-inflammatory effects. The present study investigated the neuroprotective effects of *Panax ginseng* C.A. Mayer (*P. ginseng*) after SCI. A spinal contusion was made at the T11-12 spinal cord in adult male Sprague-Dawley rats (*n* = 47) using the NYU impactor. Motor function was assessed using the Basso-Beattie-Bresnahan (BBB) score in *P. ginseng* (0.1, 0.5, 1, 3, and 5 mg/kg) or vehicle (saline) treated after SCI. We also assessed the protein expression of cyclooxygenase-2 (COX-2) and inducible nitric oxide synthase (iNOS) at the lesion site by western blot and then measured the cavity area using luxol fast blue/cresyl violet staining. *P. ginseng* treated group in SCI showed a significant improvement in locomotor function after the injury. The protein expression of COX-2 and iNOS at the lesion site and the cavity area were decreased following SCI by *P. ginseng* treatment. These results suggest that *P. ginseng* may improve the recovery of motor function after SCI which provides neuroprotection by alleviating posttraumatic inflammatory responses.

## 1. Introduction

Novel molecules from medicinal plants and botanicals play an important role in protecting the health as they have less side effects in comparison to synthetic therapeutic compounds [1]. Spinal cord injury (SCI) results in permanent loss of motor function below the injury site. At present, the available treatment alternatives are limited which can contribute to significant recovery from functional loss after SCI. Primary mechanical injury and another pathological stimulation create the injury to the secondary neuron and affect the spinal cord [2, 3]. Posttraumatic inflammation, which occurs within minutes and persists for days, is known as a possible mechanism of secondary injury after SCI [4, 5]. Previous studies have shown that nitric oxide (NO) [6], cyclooxygenase-2 (COX-2) [7], and proinflammatory cytokines [8] may contribute to the pathogenesis of secondary injury process. Therefore, management of the inflammatory reaction following SCI is important to prevent the tissue damage and protection of the cells [9].

NO production is mainly controlled by the proinflammatory enzyme inducible nitric oxide synthase (iNOS), and the expression of iNOS after SCI acts as an indicator of the inflammatory reaction that leads to secondary injury [10]. Previous studies showed that administration of iNOS inhibitor can improve motor function after SCI [11, 12]. In addition, COX-2 is induced by inflammatory stimuli and contributes to produce and release prostaglandin during inflammation. After experimental SCI using a weight drop model, COX-2 mRNA and protein production were increased in acute SCI following injury and selective inhibition of COX-2 with SC58125 improved functional recovery after SCI [13]. Recently, experimental studies using anti-inflammatory agents have shown remarkable reduction of neuronal and glial apoptosis and improvement of functional movement after SCI [14]. Nevertheless, many clinical trials with these anti-inflammatory agents have experienced limited success. Because, in many cases, there are effective when administered before SCI and are less effective at the recommended daily maximum dose even though these are administrated after injury [15]. Thus, it is necessary to develop novel anti-inflammatory agents with potent neuroprotective action and fewer side effects.

*Panax ginseng* Mayer (*P. ginseng*) is frequently used in Asian countries as a traditional medicine to treat various diseases such as atherosclerosis, hypertension, and cancer [16]. It contains two active ingredients, steroidal saponins and triterpene derivatives containing sugar moieties and more than 30 ginsenosides have been isolated from* P. ginseng* [17, 18]. These ginsenosides act as potent immune modulators by regulating cytokines [19]. Recent both* in vitro* and* in vivo* studies showed that ginseng had the effects of the neurological diseases [13] due to their anti-inflammatory, antioxidant, antiapoptosis, and immune-modulating activities [20–22]. For example,* P. ginseng* suppresses microglial activation, which was induced by lipopolysaccharide (LPS) stimulation in the mouse brain, and improves the viability of cocultured neurons with LPS-treated microglia [23]. In experimental brain injury model, intraperitoneally administered ginseng after head trauma significantly reduced the hemorrhage, infiltration, and necrosis in the traumatized region of the brain [20] and ginsenosides treatment decreased cortical contusion volume and improved the neurological deficits after traumatic brain injury [24]. In addition, ginsenoside Rg3 reduced concentration of tumor necrosis factor-*α* (TNF-*α*) and inhibited iNOS expression induced by beta amyloid (A*β*) in microglia [25]. In particular, intravenous infusion of ginsenoside Rb1 enhanced the motor recovery and reduced damage neurons after injury in both compressive SCI and ischemic brain damage [26]. Therefore, previous studies propose the therapeutic potential of ginseng to treat various neurological diseases including SCI. However, early studies related to the protective effects of ginseng against neuronal cell death have focused on a certain type of CNS injury, especially brain ischemia, and systemic studies of neuroprotective effects of ginseng on SCI are still lacking.

In the present study, we investigated the neuroprotective effect of the* P. ginseng*. This study was concerned with the pathophysiology of SCI in the presence of primary or secondary damage to the spinal cord, especially posttraumatic neuroinflammation response. Therefore, we evaluated improvement of motor function and examined the lesion size and the protein expression of iNOS and COX-2 after treatment of the* P. ginseng* in the spinal contusive rats.

## 2. Materials and Methods

### 2.1. Experimental Animals

Experiments were performed in accordance with Korea University guidelines and all animal protocols were approved by the Korea University Institutional Animal Care and Use Committee (KUIACUC-20141208-1). Fifty-four male Sprague-Dawley rats (Orient Bio Inc., Seoul, Korea) between 180 and 200 grams at the time of operation were used for this experiment. Animals were kept under a 12 h/12 h light/dark cycle (08:00–20:00) with free access to water and food* ad libitum*.

### 2.2. Extraction of the* P. ginseng*

The* P. ginseng* dried roots (400 grams) were soaked in 70% ethanol (3 liters) at room temperature for 1 day, extracted three times for 1 hour with 70% ethanol in an ultrasonic apparatus, and lyophilized by using a freeze dryer (Operon, Seoul, Korea) to produce a 70% ethanol crude extract, which was filtered through filter paper (Advantec, Toyo Roshi Kaisha, Japan). The ethanol extract was evaporated under reduced pressure by using a rotary evaporator (R-205, Büchi, Germany) and lyophilized with freeze dryer (Operon, Seoul, Korea) to produce a 70% ethanol crude extract (80 grams, yield 20%).

### 2.3. Spinal Contusion Animal Model

The spinal contusion was performed using the New York University impactor under isoflurane anesthesia (by a mixture of 4% enflurane and 95% O_2_). A laminectomy was performed at the T11 segment of the vertebra and the spinal column was stabilized by clamping the spinous processes of T10 and T12 with the alligator clips. A 10 g weight was dropped onto the spinal cord from a 12.5 mm height (*n* = 47). After the spinal cord contusion, the musculature was sutured, the skin was autoclipped, and the animals were allowed to recover from the anesthesia. A sham operation (*n* = 7) was performed using only a laminectomy of the T11 vertebra without a spinal contusion. The bladders were manually expressed twice a day until spontaneous urination returned. In order to prevent a urinary tract infection, Unasyn (ampicillin/sulbactam, 100 mg/kg, Pfizer, Seoul, Korea) was injected intraperitoneally once a day for the first 3 to 5 days. Highly absorbent bedding was used to prevent sores and infections in the paralyzed animals.

### 2.4. Experimental Design for* P. ginseng* Treatment

All rats with SCI exhibited neuronal deficits, which were characterized by paralysis in both hind paws. The rats with neurological deficits were randomly divided into a vehicle treated group (saline) and* P. ginseng* treated group.* P. ginseng* powder was dissolved in isotonic saline to the concentration of 40 mg/mL just before use. To set the dose for administration 50, 20, and 10 mg/kg of* P. ginseng (n* =* 4, resp.)* were intraperitoneally injected hour after the contusion on the basis of previous study [20]; rats with* P. ginseng at doses of 50 and 20* mg/kg died and few rats with* P. ginseng at dose of* 10 mg/kg showed the decrease of physical activity and round back position, indicating visceral pain behavior [27, 28]. Thus, in the present study, 0.1, 0.5, 1, 3, and 5 mg/kg of* P. ginseng* were used to investigate the effect of* P. ginseng* on SCI. Isotonic saline (vehicle) or 0.1, 0.5, 1, 3, and 5 mg/kg of* P. ginseng* were intraperitoneally administered an hour after the contusion in each group and that was performed once a day for 30 days.

### 2.5. Behavioral Test for Motor Function

A behavioral test for hindlimb motor function was performed at the pre- and postoperative days (POD). The test was performed on each rat 1 day prior to surgery and 1, 4, 7, 10, 13, 17, and 21 POD. The locomotor function was assessed using the BBB locomotor rating scale [29], which is based on locomotor ability following SCI in a rat model. Briefly, the BBB is a 22-point scale from 0 to 21. The 0 point indicates no observable hindlimb movement, and the 21 point indicates a consistent coordinated gait with a parallel paw placement of the hindlimbs and consistent trunk stability. The degree of motor impairment was also estimated by the combined behavioral score (CBS) [30]. This test consists of the motor function, toe spread, righting reflex, extension withdrawal reflex, placing reflex, and incline plate test. The scope of score is determined as 90 point, whereas the normal rate is 0 points and completely paralyzed rate is 90 points.

### 2.6. Western Blot

To examine whether* P. ginseng* may modulate the posttraumatic inflammatory response following SCI, the expression of iNOS and COX-2 was analyzed by western blot. The tissue samples are uninjured rats, saline injected in spinal contusive rats and 2 days after* P. ginseng* treated in spinal contusive rats. All rats were sacrificed with pentobarbital 60 mg/kg. Five rats in each group were sacrificed for this study. Rats were perfused with saline and then the injured epicenter was harvested. Protein was extracted by homogenization with protein extraction buffer (50 mM Tris-HCl (pH 7.4), 150 mM NaCl, 10 mM EDTA, 1% Triton X-100, 1% SDS, and 1% protease inhibitors) and was separated by 8% SDS-PAGE and transferred to PVDF membranes at 90 V for 2 hours on ice (Mini-PROTEAN tetra cell; Bio-rad Laboratories, Richmond, CA, USA). The membranes were blocked with 5% blocking buffer. Dissolved nonfat dried milk in TBST was used as blocking buffer. And then they were incubated with diluted anti-iNOS (1 : 1,000, milipore, Temecula, CA, USA), anti-COX2 (1 : 6,000, Abcam, Cambridge Science Park, UK), or anti-*β*-tubulin (1 : 500,000, Abcam, Cambridge Science Park, UK) in 5% blocking buffer for overnight with shaking. The membranes were washed with 0.075% TBST and incubated for an hour at room temperature with anti-rabbit IgG (Vector Lab, Burlingame, CA, USA), iNOS and COX2 (1 : 6,000), *β*-tubulin (1 : 100,000). After several washes with TBST, membranes were reacted with ECL and exposed to X-ray medical film (AGPA). Band density was analyzed by the scion image software.

### 2.7. Analysis of Lesion Volume

After the tests, the animals were randomly selected from both the* P. ginseng* and saline groups and were subjected to the comparison of histological differences in the spinal segments epicenter after contusive SCI. Histological study was conducted on 6 rats in each group 3 weeks after spinal contusion. During this period, the contused rat fully recovered motor function as described in our previous report using a rat model [1–3]. Rats were deeply anesthetized with sodium pentobarbital and perfused with heparinized saline followed by 4% paraformaldehyde in 0.1 M phosphate buffer (PB, pH 7.4). The spinal segments, including epicenter, were removed, postfixed for 6–8 hours, and stored overnight in 30% sucrose. Tissues were embedded in OCT and cut into 8 *μ*m thick longitudinal sections. The sections were treated with xylene and 95% alcohol. The slides were incubated in luxol fast blue and then counterstained with cresyl violet. To measure the level of tissue damage following SCI, size of lesion of each segment was measured by using a computer-assisted image analysis system (NIH image software). All assessments were performed in a blinded manner.

### 2.8. Statistical Analysis

All values were expressed as the mean ± standard deviation. Data were analyzed using SPSS version 13.0 software (SPSS Inc., Chicago, IL, USA). All statistical tests were evaluated at *α* level of significance of 0.05 and asterisks indicated the values (^*∗*^ *p* < 0.05). The Friedman repeated measures of analysis of the variance followed by multiple comparison tests were applied to compare the behavioral test results before and after the SCI. The iNOS and COX-2 of the lesion size were compared with* P. ginseng* treated and saline treated groups by a *t*-test.

## 3. Results

### 3.1. Effects of* P. ginseng* on Functional Motor Recovery after SCI

Prior to spinal contusion, every rat showed normal gaiting and the BBB score of rats in all groups was 21. Immediately after contusion injury, all rats in the* P. ginseng* treated groups and saline group showed paralysis of both hindlimbs, corresponding to a BBB locomotor rating scale score of 0. Progressive motor recovery was observed with hindlimb joint movement on POD 4 and the recovery proceeded relatively rapidly until POD 14. Most rats in all groups achieved maximal recovery on POD 21, which is consistent with our previous report [31].

**Figure 1:**
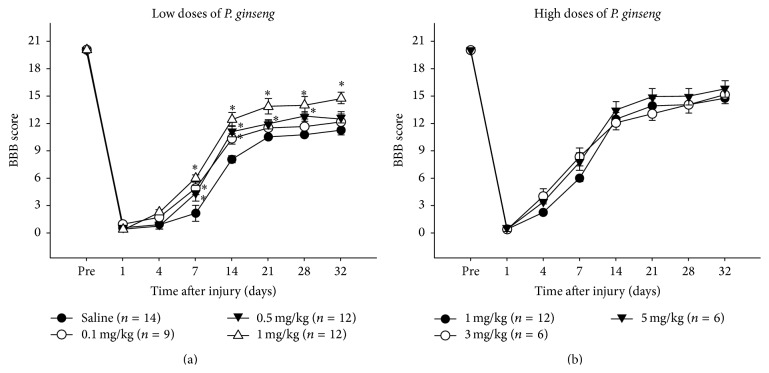
Motor recovery of hindlimb before and on days 1, 4, 7, 14, 21, 28, and 32 after SCI. (a) Low doses (0.1, 0.5, and 1 mg/kg) of* P. ginseng* and (b) high doses (1, 3, and 5 mg/kg) of* P. ginseng*.

High doses and 0.5 mg/kg of* P. ginseng* treated groups showed significantly better motor function recovery compared to the saline group throughout the entire recovery period (Figures 1(a) and 1(b)). However, there were no significant differences among the 1, 3, and 5 mg/kg of* P. ginseng* treated groups (Figure 1(b)). Functionally prominent difference in motor function between the low doses of* P. ginseng* and saline group was noticed from POD 14, even though statistically significant difference existed at POD 7 (Figure 1(a)).

In 14 days after contusion injury, the saline group scored 8.16 ± 2.75 on the BBB scale, whereas more than 1 mg/kg of* P. ginseng* treated groups (1, 3 and 5 mg/kg) scored above 12.42 ± 1.5 (Figures 1(a) and 1(b)). There was no plantar stepping and forelimb coordination on saline group. In contrast, the* P. ginseng* treated rats showed consistent weight-supported plantar steps and occasional forelimb-hindlimb coordination. In* P. ginseng* at 1, 3, and 5 mg/kg group, rats showed the significant difference up to POD 32, the last day of the study (Figure 1(b)). At this time,* P. ginseng* treated rats displayed consistent weight-supported plantar steps with consistent forelimb-hindlimb coordination, whereas the saline group showed occasional weight-supported plantar steps, with no forelimb-hindlimb coordination.

### 3.2. Effect of* P. ginseng* on COX-2 and iNOS Expression in Injured Site of SCI

**Figure 2:**
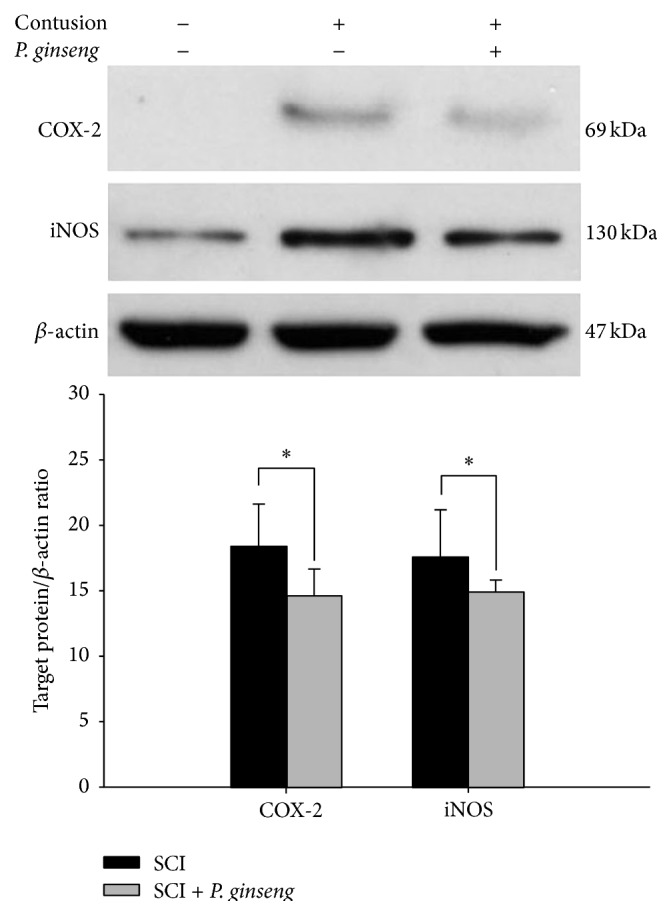
Effect of* P. ginseng* (1 mg/kg) on the COX-2 and iNOS expression. Western blot analysis of extracts of spinal cord tissue collected on 2 days after injury.* P. ginseng* treatment reduced COX-2 and iNOS levels.

To evaluate the effects of* P. ginseng* on posttraumatic inflammatory response, the protein expression of COX-2 and iNOS in the injured level of the spinal cord were investigated by western blot at 2 days after spinal contusion. Motor function recovery was statically significant differences between the high doses of* P. ginseng* treated group and saline group; however, there was no statistically significant difference in* P. ginseng* treatment expression and lesion volume after* P. ginseng* treatment in SCI rats. The expression level of COX-2 and iNOS was significantly increased in the spinal cord tissues at the lesion site of SCI with saline treated group. However, as shown in Figure 2, protein expression of COX-2 and iNOS at the injured site following SCI was significantly reduced by* P. ginseng* (1 mg/kg) treatment (*p* < 0.05).

### 3.3. Lesion Volume

**Figure 3:**
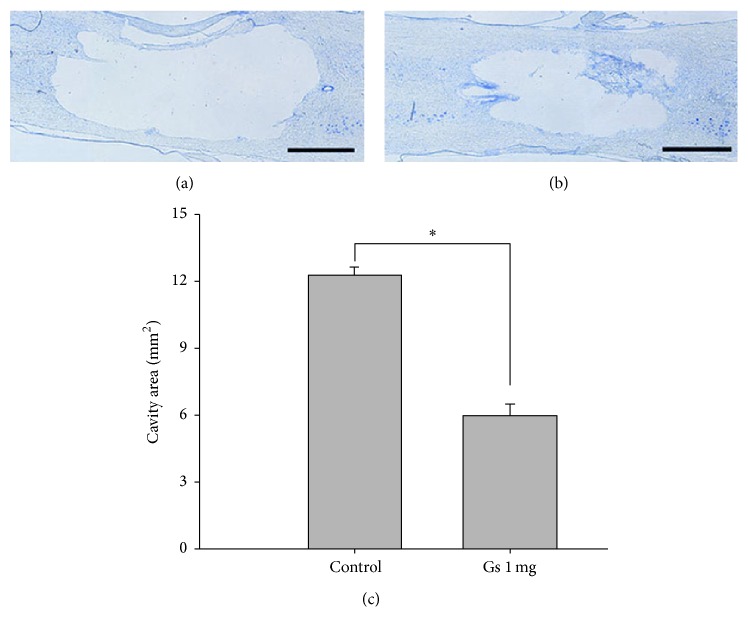
A representative section of a spinal epicenter in the* P. ginseng* (1 mg/kg) (a) and saline groups (b). Longitudinal spinal cord sections stained with luxol fast blue and cresyl violet. Scale bars: 1.5 mm.

To assess the lesion volume, the epicenter of the spinal cord was longitudinally sectioned and these slides were double-stained with luxol fast blue and cresyl violet. The lesions exhibited cystic cavitation and complete loss of normal neural tissue, including neurons and myelinated fibers. Cystic cavity appearances at the lesion site differed between* P. ginseng* treated and saline groups at 3 weeks after SCI (Figure 3(c)). The cavity extended rostrocaudally from the epicenter of the spinal cord in the saline group (Figure 3(a)). In contrast, the cavity was smaller and neural tissue was preserved at the lesion site in the 1 mg/kg of* P. ginseng* treated rats (Figure 3(b)). The cresyl violet-stained area was 5.97 ± 1.53 mm^2^ in the 1 mg/kg of* P. ginseng* treated group and was 12.23 ± 1.26 mm^2^ in the saline group, respectively (Figure 3(c)). There were statistically significant differences in the lesion areas between* P. ginseng* and saline injected rats following spinal contusion (*p* < 0.05).

## 4. Discussion

In this study, we examined the neuroprotective effects of* P. ginseng* after SCI. The present data show that* P. ginseng* decreased the protein expression of iNOS and COX-2 following spinal contusion, thereby leading to enhanced functional recovery SCI. Furthermore, lesion size was significantly smaller in* P. ginseng* treated than in saline injected rats. Thus, this study shows that* P. ginseng* has neuroprotective and anti-inflammatory effects on SCI.

In this study, we examined effects of* P. ginseng* on functional recovery up to POD 32 after SCI. The present results show that* P. ginseng* treatment enhanced the improvement of locomotor recovery as compared to saline group. As a result, the significant difference in locomotor function between the* P. ginseng* treated group and the saline injected group was shown at the higher dose of* P. ginseng* (5, 3, and 1 mg/kg) from POD 7. However, functionally prominent difference in locomotor function between* P. ginseng* treated group and saline group appeared from POD 14. The difference in hindlimb function quality between the* P. ginseng* treated group and saline group was huge at this time point, although the score difference on BBB expressed, it may not be great.* P. ginseng* treated group,* except* 0.1 mg/kg treated group, displayed nearly consistent plantar stepping with consistent forelimb-hindlimb coordination, whereas saline group merely placed their limbs in a weighted fashion. In this study, motor function recovery plateaued at 3 weeks after SCI as our previous reports [1–3]. Interestingly,* P. ginseng* treated rats at higher dose showed the prominent motor recovery until 32 POD, the last day of this study. In the present study, there was no significant difference in the degree of motor recovery among three higher doses* P. ginseng* treated groups (5, 3, and 1 mg/kg) during the entire recovery periods. In our study, the doses of* P. ginseng* (0.1, 0.5, 1, 3, and 5 mg/kg) were used and* P. ginseng* was intraperitoneally applied after spinal contusion. Recently, protective effects of ginseng have been demonstrated, for instance, by reducing the necrosis in the neuronal region of the brain and improving the motor recovery by ginseng extract or ginsenosides in experimental brain injury. Previous research revealed the neuroprotective effect of ginseng by using higher doses (above 20 mg/kg, intraperitoneal administration) compared to doses used in this study [20]. This disparity can be explained in part by the fact that research has been conducted by using various ginseng extract methods and different administration route.

Many studies have shown the involvement of proinflammatory mediators in cell death after SCI [32, 33]. After SCI, microglia is activated and mediates the acute neuroinflammatory response by releasing proinflammatory cytokines [2, 34]. Increased inflammatory cytokines contribute to further exacerbate the neuroinflammatory pathways which may decrease the potential for functional plasticity in the nervous system after injury [35]. Particularly, iNOS and COX-2 are rapidly upregulated after SCI [36]. Our data showed that* P. ginseng* treatment decreased the protein expression of iNOS and COX-2 at the lesion site following SCI. The present results are in agreement with previous reports showing anti-inflammatory effects of ginseng in beta amyloid- (A*β*-) activated microglia cell [25]. It has been well known that SCI leads to a progressive series of degenerative processes induced by the original insult to the spinal cord. These secondary neuroinflammatory processes, which contribute to progressive tissue loss and cavitation at the injury site, are a major cause of motor dysfunction [37]. Our findings are consistent with previous studies where inhibition of the inflammatory reaction produces beneficial effects after SCI. Anti-inflammatory drugs, such as minocycline, improved functional recovery and reduced cell death after traumatic SCI in rats [38]. Our results are consistent with earlier studies on traumatic insult to the brain [24]. Intraperitoneally injected ginseng reduced the infiltration and necrosis in lesion area after brain injury in rats.* P. ginseng* treatment also decreased cortical contusion volume and improved the neurological deficits after traumatic brain injury. Considered together, the enhancement of motor function and the reduction of cell death after SCI in* P. ginseng* treated rats may be partially mediated by the anti-inflammatory effect of* P. ginseng*. No sign of side effects such as loss of body weight was observed at concentrations of* P. ginseng* extract used in the present study. We did not examine the anti-inflammatory effects of active compounds isolated from* P. ginseng* extract in this study. Thus, further studies on active compounds isolated from a GTS extract in their anti-inflammatory effects after SCI are needed.

## 5. Conclusion

In summary, we have investigated the neuroprotective effect of* P. ginseng* as a potential therapeutic agent after SCI. Our present results demonstrated that* P. ginseng* results in a remarkable increase in functional outcome and a decrease in lesion size by inhibition of inflammatory reaction after SCI. The present report suggests the ideas that* P. ginseng* has a neuroprotective effect after SCI and* P. ginseng* can be used as a candidate for the development of a therapeutic agent such as for human SCI.
